# Vapor Sorption and Halogen-Bond-Induced Solid-Form Rearrangement of a Porous Pharmaceutical

**DOI:** 10.1021/acs.cgd.2c01464

**Published:** 2023-02-28

**Authors:** Jessica L. Andrews, Dmitry S. Yufit, James F. McCabe, Mark A. Fox, Jonathan W. Steed

**Affiliations:** † Department of Chemistry, Durham University, South Road, Durham DH1 3LE, U.K.; ‡ Pharmaceutical Sciences, R&D, AstraZeneca, Charter Way, Silk Road Business Park, Macclesfield SK10 2NA, U.K.

## Abstract

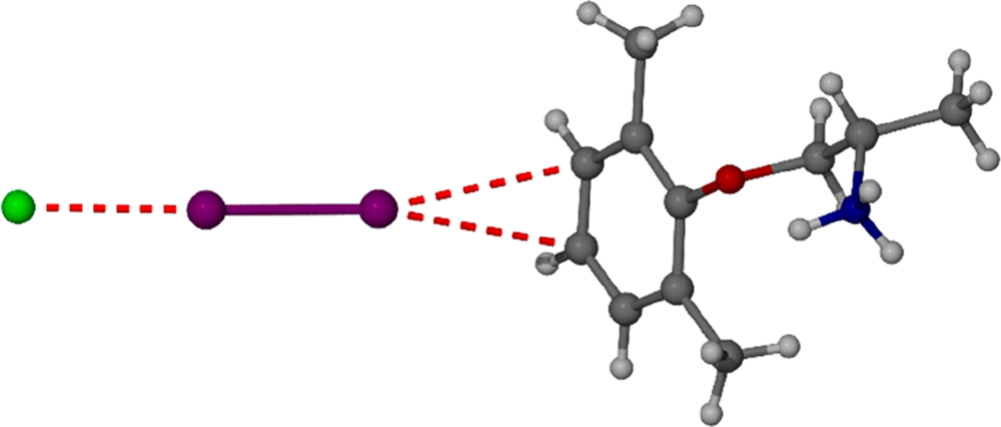

A porous, nonsolvated polymorph of the voltage-gated sodium channel blocker mexiletine hydrochloride absorbs iodine vapor to give a pharmaceutical cocrystal incorporating an I_2_Cl^–^ anion that forms a halogen−π interaction with the mexiletine cations. The most thermodynamically stable form of the compound does not absorb iodine. This example shows that vapor sorption is a potentially useful and underused tool for bringing about changes in pharmaceutical solid form as part of a solid form screening protocol.

## Abstract 2

A porous, nonsolvated polymorph of the voltage-gated sodium channel blocker mexiletine hydrochloride absorbs iodine vapor to give a pharmaceutical cocrystal incorporating an I_2_Cl^−^ anion that forms a halogen−π interaction with the mexiletine cations.

## Introduction

Understanding and controlling the solid form landscape of pharmaceuticals is a key aspect of drug preformulation and a requirement of any new drug application.^1,2^ Techniques to crystallize molecular compounds are well understood and generally take the form of solution-based evaporation and cooling methods along with other techniques such as heating, sublimation, mechanochemistry, and desolvation.^3−5^ In conjunction with increasingly powerful computational prediction approaches,^6−8^ these methods form the basis of routine pharmaceutical solid form screening.^9,10^ Frequently, however, many more polymorphs are calculated in an accessible energy range using computational crystal structure prediction (CSP) methods that have been discovered experimentally.^11^ As a result, there is considerable interest in novel crystallization approaches that might allow the discovery of solid forms that do not crystallize using conventional solution-based methods. The recent reports of the 13th and 14th forms of the highly polymorphic olanzapine precursor ROY discovered using heteroseeding^12^ and nanodroplet-based^13^ techniques illustrate that hitherto unknown solid forms of compounds thought to have been comprehensively studied can be realized experimentally with unconventional crystallization methods. Novel crystallization techniques such as supramolecular gel-phase crystallization,^14−16^ microemulsions,^17,18^ confinement,^19^ and high-pressure crystallization have all resulted in the discovery of new solid forms, in some cases predicted computationally in advance.^20,21^ One underexplored polymorph discovery tool is the use of vapor sorption to bring about solid form transformation. While vapor sorption by metal organic frameworks and noncovalent assemblies is a topical and useful process,^22−24^ it is rarely applied in a pharmaceutical context. In a key 2011 report, carbon dioxide gas-induced transformations of clarithromycin and lansoprazole were shown to result in conversion of solvate forms to commercially useful nonsolvated polymorphs.^25^ In a nonpharmaceutical context, absorption of dihalogen vapor by nonporous onium ion salts is known to result in solid form transformations by direct solid–vapor reaction to give trihalide salts in which a dihalogen such as I_2_ is absorbed by a chloride salt of 1,6-bis(trimethylammonium)hexane to resulting in halogen-bonded anions such as I_2_Cl^–^.^26^ While the clarithromycin and lansoprazole example involves neutral pharmaceutical molecules, many drugs are used in salt form, and among pharmaceutical salts chloride is by far the most common counteranion.^27,28^ In the present work, we combine the concepts of vapor-sorption-based solid-form transformation and halogen bonding to halide salts to attempt to bring about solid form changes in a pharmaceutical chloride salt, mexiletine hydrochloride. The chloride anion in examples such as this might be expected to halogen bond strongly with iodine vapor.

**Figure 1:**
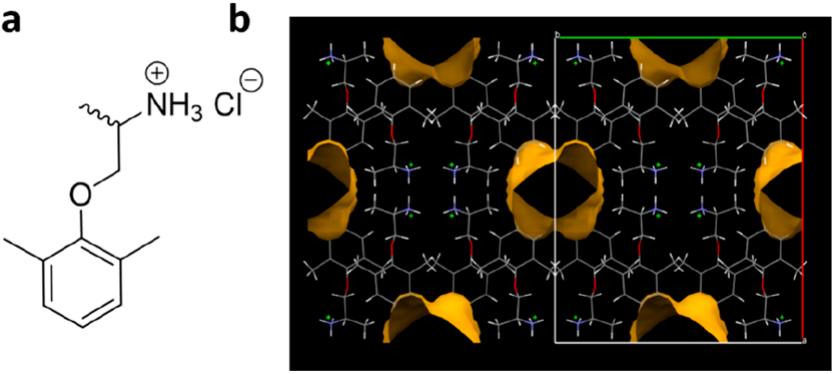
(a) Chemical structure of mexiletine hydrochloride. (b) Void channels in the solvent-free Type A phase.^30^

Mexiletine is a nonselective voltage-gated sodium channel blocker used to treat abnormal heart rhythms, chronic pain, and some causes of muscle stiffness.^29^ It has five important solid form classes.^30^ Forms 1,^31^ 2, and 3^32^ are mutually enantiotropically related anhydrous polymorphs, and there are two extensive related families of metastable channel solvates termed Types A and B.^30^ The polymorphic nature of mexiletine allows comparison of vapor response as a function of different starting structures, and it is particularly interesting that solvate families of types A and B also exist as solvent-free phases with empty channels that might prove porous. Thus, the empty type A and B solvates represent the fourth and fifth mexiletine hydrochloride polymorphs. In the case of the solvent-free Type A form, the single-crystal structure is known^30^ and the voids comprise some 444 Å^3^ or 15.8% of the unit cell volume, Figure 1.

## Results and Discussion

**Figure 2:**
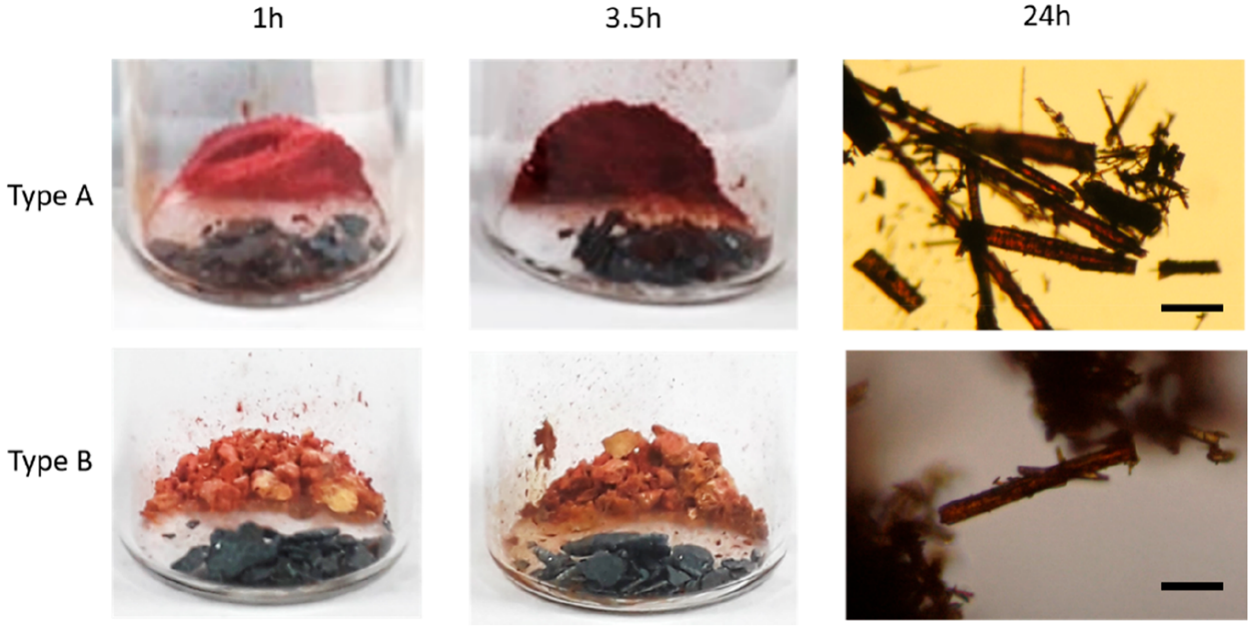
Gas sorption experiments showing the color change of the Type A and B solvent-free forms after 1 and 3.5 h of exposure to iodine vapor. After 24 h the crystals were visually examined under a microscope (scale bar 100 μm).

**Figure 3:**
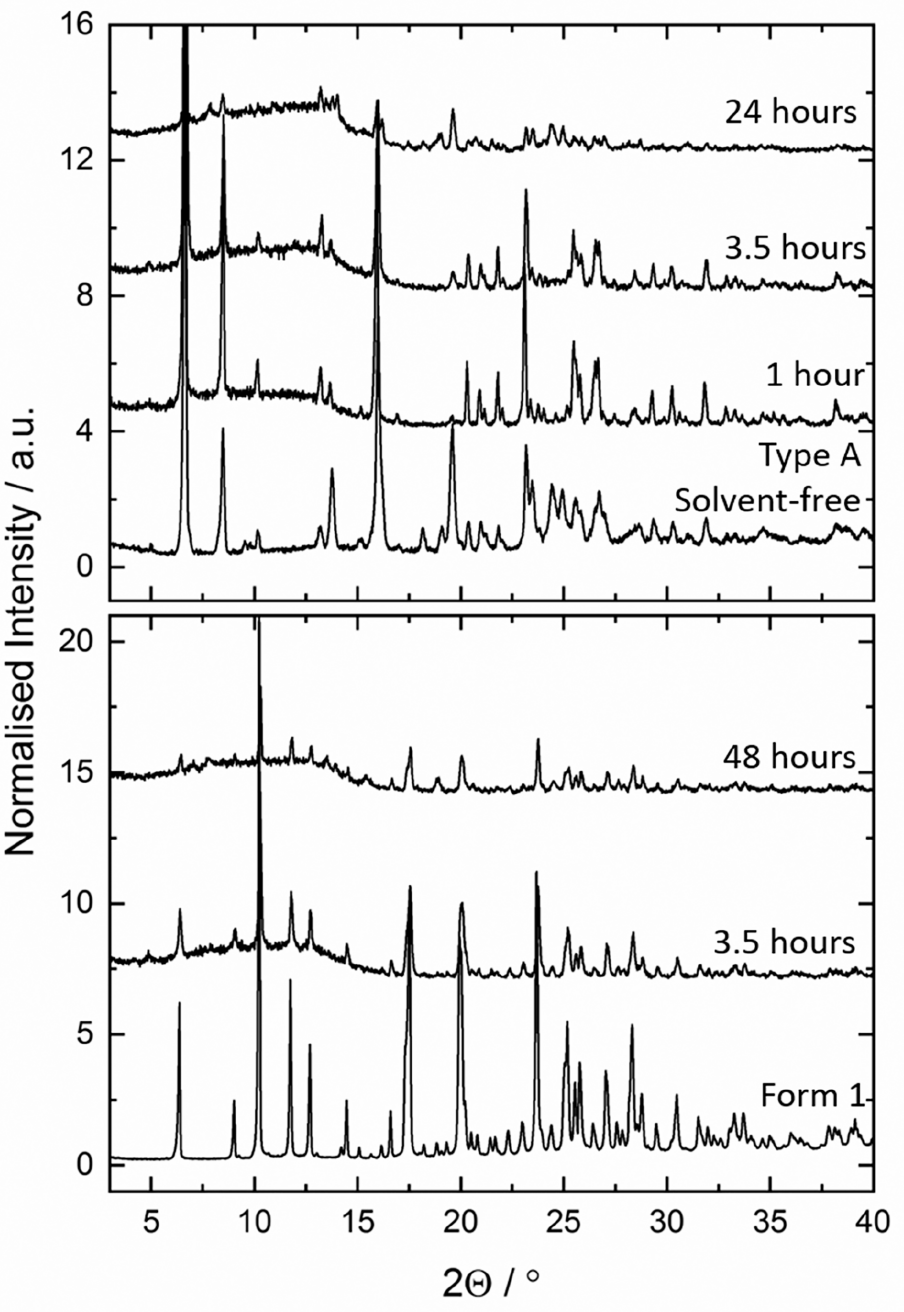
XRPD patterns of the solvent-free Type A structure and Form 1, following exposure to iodine vapor for different lengths of time.

Iodine vapor sorption experiments^33^ were carried out on both the close-packed room-temperature stable polymorph Form 1 and the solvent-free Type A and B phases of mexiletine hydrochloride. Iodine is particularly advantageous due to its characteristic color, which provides a visual indication of whether the gas has been absorbed. Tests were carried out by placing a crystalline sample of all three forms into a sealed vial close to but not touching a similar mass of solid iodine (Figures 2 and 3). The experiments were carried out at room temperature, allowing the iodine to sublime gradually, and crystals were monitored visually for color changes and by X-ray powder diffraction (XRPD). The mexiletine and iodine powders were placed on opposite sides of the same vial. This meant that the front of the drug powder was exposed more directly to the iodine vapor than the back, which led to uneven iodine adsorption in the early stages of the experiment. However, the two empty channel forms both became uniformly colored within 1 h of iodine exposure, and in each case, the mexiletine-containing powder was thoroughly mixed before XRPD analysis.

When exposed to iodine vapor, crystals of the solvent-free Type A form begin to change color immediately and darken significantly over time from light pink, to purple, to brown. When viewed under a microscope, the Type A crystals appear uniformly colored, implying that iodine permeates the channels in the structure, rather than simply adsorbing onto the crystal surface. Similar results were observed for the solvent-free Type B form, which rapidly darkened in color when exposed to the vapor (Figure 2). While individual Type B crystals became uniformly brown after 24 h, the bulk sample remained patchy in color and did not absorb iodine as efficiently as Type A, which is likely due to a polymorphic transition to Form 1 over time. These observations indicate that both solvent-free structures are porous and suggest that the iodine molecules diffuse into the channels in the drug framework. However, there is also evidence for some cracking and pseudomorphosis, implying degradation of crystallinity or a phase change. In contrast, Form 1 showed a much slower color change from white to light brown. The color was more intense on the edge of the powder than in the middle, which suggests this form is not permeable to iodine and the sample only undergoes some limited surface sorption (Supporting Information, Figure S2). If removed from the iodine vapor and stored in air, the purple color was lost rapidly from all samples, which suggests that the iodine molecules are only loosely bound to the drug structure, mirroring the behavior of other guests such as organic solvents, bound within the channel solvates.

The XRPD patterns of the Type A and Form 1 samples developed a significant amorphous background with increased exposure to iodine vapor, signifying a decrease in crystallinity (Figure 3). Useful XRPD data was not obtained on the Type B material because its XRPD pattern already contains a significant amorphous background, as it is crystallized by fast cooling. The XRPD pattern of Form 1 remained unchanged with exposure to iodine, except for the reduced crystallinity. However, significant changes were observed for the solvent-free Type A form. With a short exposure time the key peaks characteristic of the Type A structure remained. However, with a longer exposure time, a mixture of Form A and a new crystalline material was produced. The XRPD pattern of this new phase does not match either the Type A solvates or Form 1.

In order to attempt to prepare a more crystalline sample of this new phase, the solution cocrystallization of mexiletine and iodine was carried out by vapor diffusion of hexane into an equimolar solution of mexiletine and iodine in dichloromethane. This resulted in crystals that were characterized by single-crystal X-ray crystallography as an approximately 2:1:1 cocrystal solvate containing mexiletine hydrochloride, I_2_, and CH_2_Cl_2_, respectively. In the crystal studied, the iodine is disordered across two sites with occupancies 0.82 and 0.07, total 0.89. The remaining 0.11 site occupancy is filled by a molecule of CH_2_Cl_2_ giving the precise stoichiometry as (mexiletine HCl)_2_(I_2_)_0.82_(CH_2_Cl_2_)_1.11_. Crystallographic information is given in the Supporting Information. Given the bioactivity of mexiletine and the role of iodine as an antiseptic,^34,35^ this material is notionally a drug–drug cocrystal (or more precisely, a drug–drug ionic cocrystal solvate), although given that iodine is generally applied topically, a practical application of such a comedication is unlikely.^36,37^

**Figure 4:**
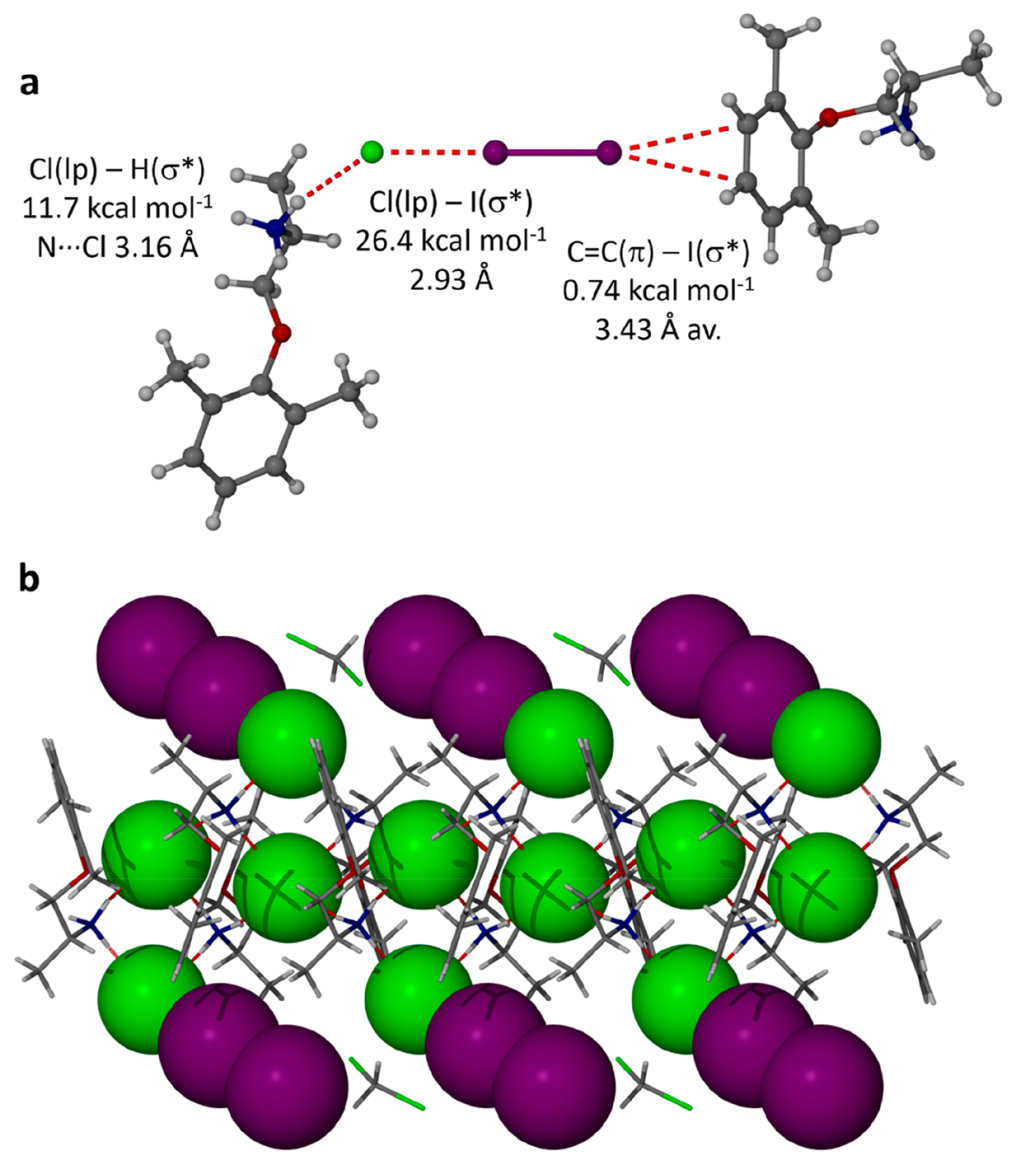
(a) Hydrogen bonding, halogen bonding, and iodine−π interactions in the I_2_Cl^–^ anion in (mexiletine hydrochloride)_2_·I_2_·CH_2_Cl_2_. (b) 1D hydrogen bond chain based NH_3_^+^···Cl^–^ hydrogen bonding flanked by the I_2_Cl^–^ anions. Space between the iodine units is filled by disordered CH_2_Cl_2_. The next layer interacts with the iodine by an iodine−π interaction.

In the iodine cocrystal solvate the dominant iodine site forms a halogen bond with one of the two independent chloride anions to give an I_2_Cl^–^ anion with an I···Cl distance of 2.93 Å (Figure 4a). The other end of the I_2_ molecule forms an iodine-π interaction,^38^ in which one C=C double bond of an aromatic ring acts as an electron donor to the iodine σ* orbital. The I···C distances are quite short at 3.42 and 3.44 Å, with only the iodine complex of coronene being significantly shorter at 3.17 and 3.37 Å, respectively.^39^ The I_2_Cl^–^ anions flank a 1D hydrogen-bonded chain structure based on NH_3_^+^···Cl^–^ hydrogen bonding (Figure 4b). These interactions were verified by natural bond orbital (NBO) calculations (see the Supporting Information for details), which indicated that the halogen bond is significantly stronger than the NH···Cl^–^ hydrogen bond (26.39 vs 11.65 kcal mol^–1^). The iodine−π interaction is present but very weak in comparison at 0.74 kcal mol^–1^. The central chloride anions accept four hydrogen bonds in a roughly tetrahedral array from the NH_3_^+^ groups (N···Cl distances are all around 3.2 Å), while the chloride ion that forms part of the I_2_Cl^–^ anion accepts two shorter NH···Cl interactions (3.18 Å av) as well as a long CH···Cl interaction from the dichloromethane. The iodine molecule is disordered with a second position present in just 7% of unit cells and is close to the primary position and forms similar interactions. Interestingly, the iodine cocrystal is essentially isostructural with Form 1 which has the same 1D hydrogen-bonded tape structure based on two independent chloride ions.^30,31^ In Form 1 the two independent chloride ions form four and two NH···Cl interactions, as in the iodine structure. As a result, in Form 1 one of the chloride ions is exposed in a way that should be amenable to iodine binding and in the iodine cocrystal the iodine fits between the strands resulting in a very similar unit cell to Form 1. The crystallographic *b* axis is expanded from 10.60 Å in Form 1 to 13.66 Å to accommodate the added iodine, while the *a* and *c* axes are almost unchanged. The two mexiletine molecules in the asymmetric unit of both Form 1 and the iodine adduct have identical conformations with an RMSD of only 0.040 Å when the two molecules are compared. It is surprising, therefore, that iodine does not interact more readily with the Form 1 crystals, and this must arise from its nonporous structure.

**Figure 5:**
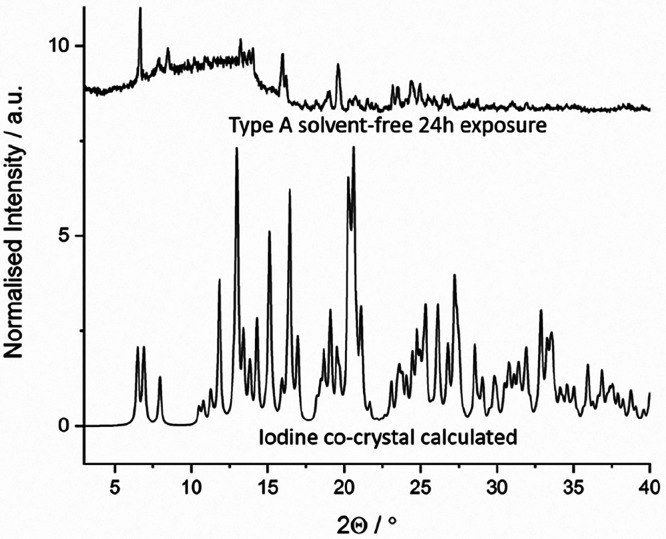
XRPD pattern of the solvent-free Type A form, exposed to iodine vapor for 24 h, compared to the calculated XRPD pattern of the mexiletine-iodine cocrystal solvate.

The calculated XRPD pattern of the iodine cocrystal solvate has some features in common with that of the new phase that forms upon exposure of the solvent-free Type A structure to iodine for 24 h, for example, a new peak at 8.08° 2θ (Figure 5). While the iodine vapor diffusion is not carried out in the presence of dichloromethane, it is likely that a range of volatile species including oxygen, nitrogen, or CO_2_ from air^40^ can occupy the lattice gap between the iodine units in the iodine cocrystal. We conclude that exposure of the solvent-free Type A structure results in rapid iodine absorption to give a transient Type A iodine solvate, followed by very significant rearrangement to give a new iodine drug–drug cocrystal phase along with considerable amorphization. The structure of the cocrystal is very different to both Types A and B solvates and the high degree of molecular reorganization required to change between these two forms is likely responsible for the amorphization of these samples.

In view of the CO_2_-induced transformation of clarithromycin and lansoprazole solvate forms to commercially useful nonsolvated polymorphs^25^ we also exposed the desolvated Type A mexiletine hydrochloride to CO_2_ gas by means of placing a chip of solid CO_2_ next to a sample of the material in a loosely capped vial. This indeed resulted in the transformation of the sample from porous Type A to nonsolvated Form 1 (Supporting Information, Figure S1). While this transformation is not commercially useful in the case of mexiletine, it confirms the novel role of gases in bringing about solid form transformations. It is possible that similar treatment with supercritical fluids such as scCO_2_ may be useful in this regard, replacing organic slurry/solvent-mediated transformations with a greener alternative, and there exist a number of reports discussing the use of supercritical media in pharmaceutical solid form studies.^41−43^

## Conclusions

In conclusion, a porous metastable desolvated form of mexiletine hydrochloride undergoes vapor sorption of iodine into the empty channels followed by rearrangement to a new drug–drug ionic cocrystal that is closely related to the most thermodynamically stable polymorph of the drug at room temperature, Form 1. Interestingly, Form 1 itself does not directly incorporate iodine into its lattice. The new cocrystal has an unusual structure incorporating both halogen bonding and weak halogen−π interactions as well as conventional NH···Cl^–^ hydrogen bonds. More generally, this method shows that vapor sorption represents a novel and underused way of bringing about changes in the pharmaceutical solid form as part of the arsenal of polymorph screening techniques.

## Supporting Information Available

Single-crystal and powder X-ray crystallographic data and details of the NBO calculations (PDF)
