# Supplementary material for: Vapor Sorption and Halogen-Bond-Induced Solid-Form Rearrangement of a Porous Pharmaceutical
Source: Cryst Growth Des. 2023 Feb 28;23(4):2628–33. doi: 10.1021/acs.cgd.2c01464 (PMC10080649; doi:10.1021/acs.cgd.2c01464)
Supplement: Supplementary file 1 — cg2c01464_si_001.pdf [file cg2c01464_si_001.pdf]

Vapor Sorption and Halogen Bond Induced Solid Form Rearrangement of a Porous Pharmaceutical

Electronic Supplementary Information

Jessica L. Andrews,[a] Dmitry S. Yufit,[a] James F. McCabe[b], Mark A. Fox and Jonathan W. Steed*[a]

a) *Durham University, Department of Chemistry, Lower Mountjoy, Stockton Road, Durham, DH1 3LE, UK. Email: jon.steed@durham.ac.uk*

b) *Pharmaceutical Sciences, R&D, AstraZeneca, Macclesfield, UK.*

Single Crystal X-ray Crystallography

Table S1: Comparative unit cell information for Form 1, desolvated type A, and the iodine co-crystal solvate of mexiletine hydrochloride

	**Form 1**[1]	**Desolvated Type A**[2]	**Iodine co-crystals solvate**
**Space group**	$P\bar{1}$	*Pbcn*	$P\bar{1}$

***a*/Å**	8.796(15)	20.8570(2)	8.7220(9)
***b*/Å**	10.601(18)	17.3783(18)	13.6572(14)
***c*/Å**	14.229(24)	7.5648(8)	14.6845(14)
***α*/°**	78.74(13)	90	70.816(4)
***β*/°**	79.89(14)	90	76.280(4)
***γ*/°**	68.69(12)	90	78.824(4)
**Volume/Å^3**	1204.327	2741.9(5)	1592.1(3)

Table S2: Crystallographic information for the co-crystal solvate containing mexiletine hydrochloride, iodine, and DCM.

Crystallisation conditions	Vapour diffusion of hexane into an equimolar solution of mexiletine and iodine in DCM
Empirical formula	$C_{11.55}H_{19.11}Cl_{2.11}I_{0.89}NO$
Formula weight	375.79
Temperature/K	120.00
Crystal system	triclinic
Space group	P-1
a/Å	8.7220(9)
b/Å	13.6572(14)
c/Å	14.6845(14)
α/°	70.816(4)
β/°	76.280(4)
γ/°	78.824(4)
Volume/Å^3	1592.1(3)
Z	4

ρ_{calc}g/cm^3	1.568
μ/mm^{-1}	2.137
F(000)	746.0
Crystal size/mm^3	0.079 × 0.055 × 0.042
Radiation	Mo Kα (λ = 0.71073)
2Θ range for data collection/°	4.846 to 54.994
Index ranges	-11 ≤ h ≤ 11, -17 ≤ k ≤ 17, -19 ≤ l ≤ 19
Reflections collected	29052
Independent reflections	7312 [R_{int} = 0.1083, R_{sigma} = 0.1221]
Data/restraints/parameters	7312/6/356
Goodness-of-fit on F^2	0.985
Final R indexes [I>=2σ (I)]	R_1 = 0.0510, wR_2 = 0.0976
Final R indexes [all data]	R_1 = 0.1095, wR_2 = 0.1126
Largest diff. peak/hole / e Å^{-3}	1.21/-1.01

The X-ray single crystal data were collected using Mo-Kα radiation (λ =0.71073Å) on a Bruker D8Venture (Photon100 CMOS detector, IμS-microsource, focusing mirrors) 3-circle diffractometer equipped with a Cryostream (Oxford Cryosystems) open-flow nitrogen cryostat at the temperature 120.0(2)K. The structure was solved by direct method and refined by full-matrix least squares on F^2 for all data using Olex2[3] and SHELXTL[4] software. All non-hydrogen atoms were refined in anisotropic approximation, iodine atoms of a minor component of disordered iodine molecule (fixed SOF = 0.03) were refined isotropically. The hydrogen atoms were placed in the calculated positions and refined in riding mode. Crystal data and parameters of refinement are listed in Table S2. Crystallographic data for the structure have been deposited with the Cambridge Crystallographic Data Centre as supplementary publication CCDC-2204560.

Powder Diffraction Data

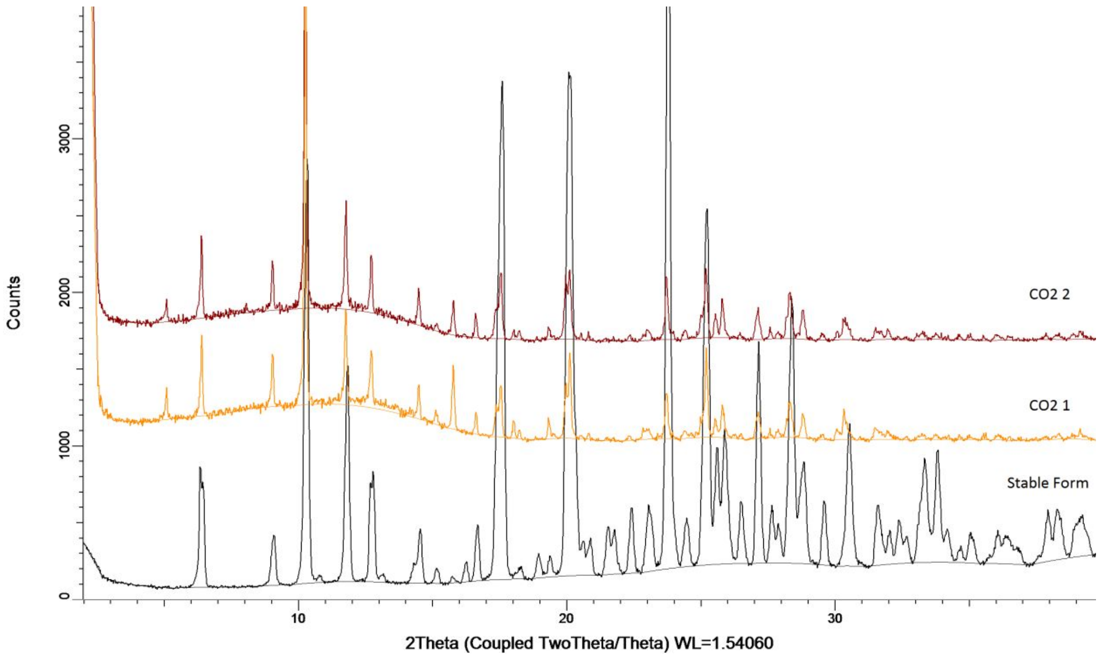

Fig. S1. Comparison of the XRPD patterns of desolvated Form A mexiletine hydrochloride after exposure to CO_2 gas at ambient pressure (two repeats) with the stable Form 1.

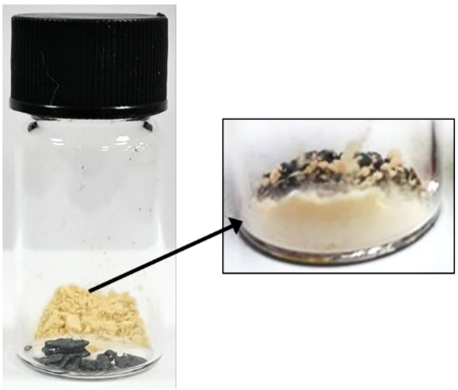

Fig. S2. Gas sorption experiment showing the colour change of Form 1 after 3.5 hours of exposure to iodine vapour. The inset image is viewed from the back, showing that the colour change is localised to the surface of the powder.

NBO Calculations

Natural bond orbital (NBO)[5-6] calculations were carried out on model geometries at M06-2X[7]/def2-TZVP[8-9] using the NBO 3.1 program within the Gaussian 16 package.[10] The model geometries with three different iodine positions were generated from the X-ray crystal structure of the co-crystal solvate containing mexiletine hydrochloride, iodine and DCM. The hybrid-DFT functional M06-2X was selected here as in a study M06-2X correctly estimated the halogen bond energies between neutral and anionic species of halogens whereas other functionals employed overestimated the halogen bond energies.[11] The large def2-TZVP basis set was applied to all atoms here.

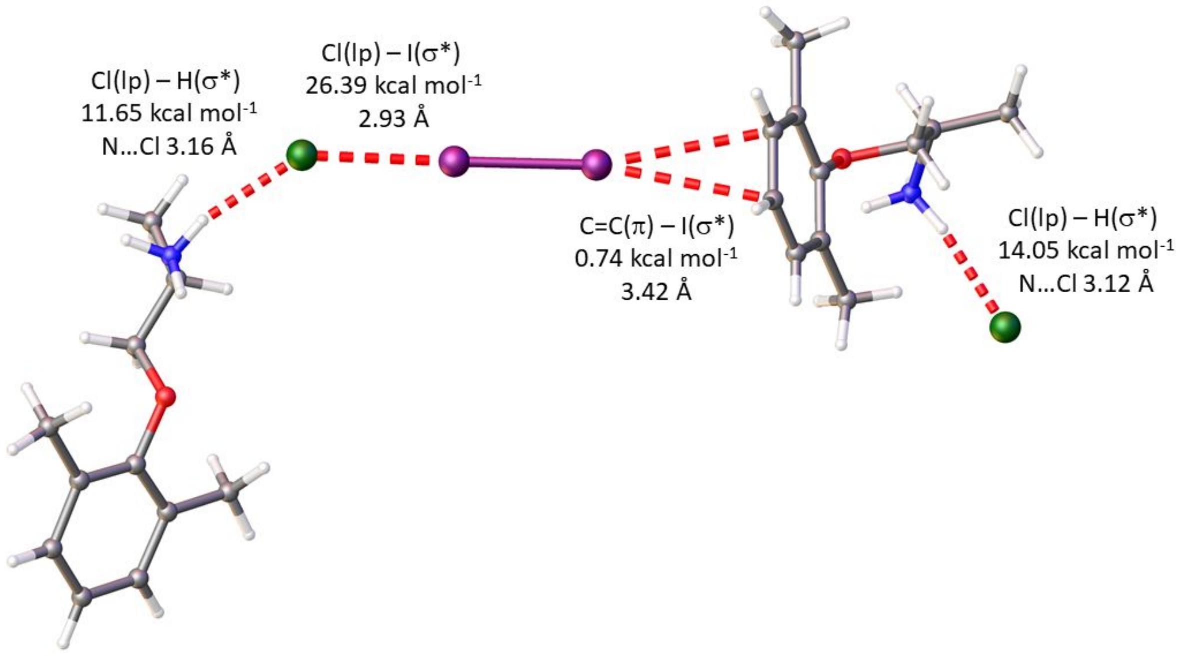

Fig. S3. Hydrogen bonding, halogen bonding and iodine-π interactions in the I_2Cl^- anion with the iodine molecule at the major occupancy position (0.82) in the co-crystal solvate containing mexiletine hydrochloride, iodine and DCM. Nature and energies of interactions were determined by NBO calculations.

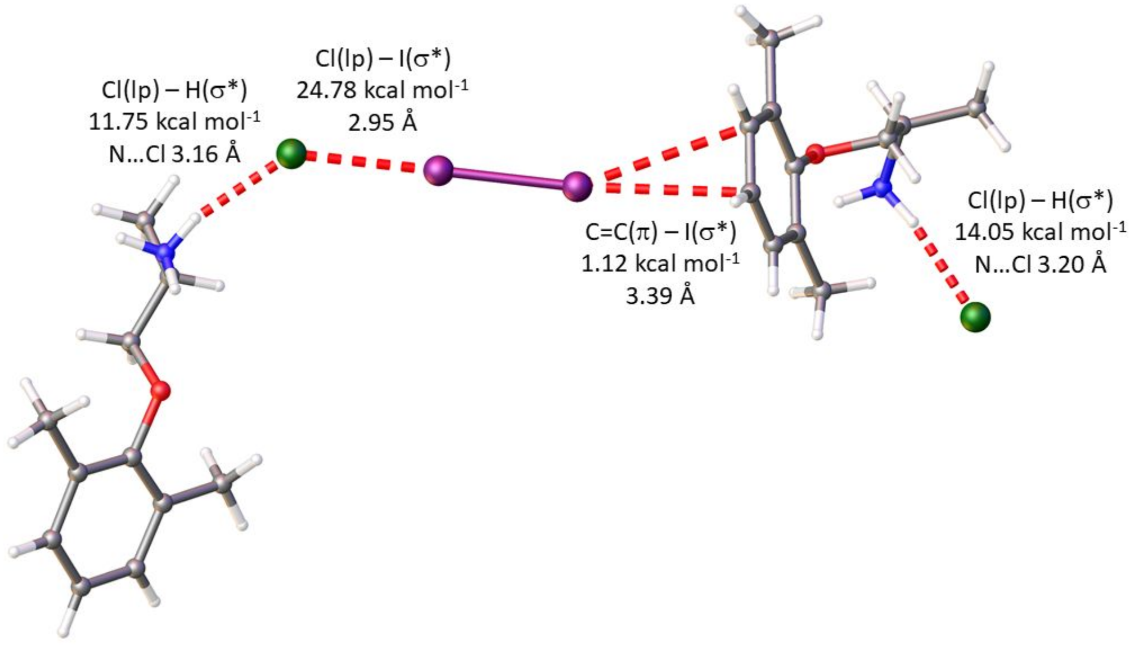

Fig. S4. Hydrogen bonding, halogen bonding and iodine-π interactions in the I_2Cl^- anion with the iodine molecule at a minor occupancy position (0.03) in the co-crystal solvate containing mexiletine hydrochloride, iodine and DCM.

References

(1) Sivy, J.; Kettmann, V.; Fresova, E., Structure of 1-(2,6-dimethylphenoxy)-2-propanamine hydrochloride. *Acta Crystallogr. Sect. C* **1991**, *47*, 2695-2696.

(2) Andrews, J. L.; Lill, S. O. N.; Freitag-Pohl, S.; Apperley, D. C.; Yufit, D. S.; Batsanov, A. S.; Mulvee, M. T.; Edkins, K.; McCabe, J. F.; Berry, D. J.; Probert, M. R.; Steed, J. W., Derisking the Polymorph Landscape: The Complex Polymorphism of Mexiletine Hydrochloride. *Cryst. Growth Des.* **2021**, *21*, 7150-7167.

(3) Dolomanov, O. V.; Bourhis, L. J.; Gildea, R. J.; Howard, J. A. K.; Puschmann, H., OLEX2: a complete structure solution, refinement and analysis program. *J. Appl. Crystallogr.* **2009**, *42*, 339-341.

(4) Sheldrick, G. M., A short history of SHELX. *Acta Crystallogr. Sect. A* **2008**, *64*, 112-122.

(5) Foster, J. P.; Weinhold, F., Natural hybrid orbitals. *J. Am. Chem. Soc.* **1980**, *102*, 7211-7218.

(6) Reed, A. E.; Weinhold, F., Natural bond orbital analysis of near-Hartree–Fock water dimer. *J. Chem. Phys.* **1983**, *78*, 4066-4073.

(7) Zhao, Y.; Truhlar, D. G., The M06 suite of density functionals for main group thermochemistry, thermochemical kinetics, noncovalent interactions, excited states, and transition elements: two new functionals and systematic testing of four M06-class functionals and 12 other functionals. *Theoretical Chemistry Accounts* **2008**, *120*, 215-241.

(8) Weigend, F.; Ahlrichs, R., Balanced basis sets of split valence, triple zeta valence and quadruple zeta valence quality for H to Rn: Design and assessment of accuracy. *PCCP Phys. Chem. Chem. Phys.* **2005**, *7*, 3297-3305.

(9) Weigend, F., Accurate Coulomb-fitting basis sets for H to Rn. *PCCP Phys. Chem. Chem. Phys.* **2006**, *8*, 1057-1065.

(10) Frisch, M. J.; Trucks, G. W.; Schlegel, H. B.; Scuseria, G. E.; Robb, M. A.; Cheeseman, J. R.; Scalmani, G.; Barone, V.; Petersson, G. A.; Nakatsuji, H.; Li, X.; Caricato, M.; Marenich, A. V.; Bloino, J.; Janesko, B. G.; Gomperts, R.; Mennucci, B.; Hratchian, H. P.; Ortiz, J. V.; Izmaylov, A. F.; Sonnenberg, J. L.; Williams-Young, D.; Ding, F.; Lipparini, F.; Egidi, F.; Goings, J.; Peng, B.; Petrone, A.; Henderson, T.; Ranasinghe, D.; Zakrzewski, V. G.; Gao, J.; Rega, N.; Zheng, G.; Liang, W.; Hada, M.; Ehara, M.; Toyota, K.; Fukuda, R.; Hasegawa, J.; Ishida, M.; Nakajima, T.; Honda, Y.;

Kitao, O.; Nakai, H.; Vreven, T.; Throssell, K.; Montgomery Jr., J. A.; Peralta, J. E.; Ogliaro, F.; Bearpark, M. J.; Heyd, J. J.; Brothers, E. N.; Kudin, K. N.; Staroverov, V. N.; Keith, T. A.; Kobayashi, R.; Normand, J.; Raghavachari, K.; Rendell, A. P.; Burant, J. C.; Iyengar, S. S.; Tomasi, J.; Cossi, M.; Millam, J. M.; Klene, M.; Adamo, C.; Cammi, R.; Ochterski, J. W.; Martin, R. L.; Morokuma, K.; Farkas, O.; Foresman, J. B.; Fox, D. J. *Gaussian 16, Revision B.01*, Gaussian Inc.: Pittsburgh (PA), 2016.

(11) Bauzá, A.; Alkorta, I.; Frontera, A.; Elguero, J., On the Reliability of Pure and Hybrid DFT Methods for the Evaluation of Halogen, Chalcogen, and Pnicogen Bonds Involving Anionic and Neutral Electron Donors. *J. Chem. Theor. Comput.* **2013**, *9*, 5201-5210.
